# Association of hearing loss with depression, anxiety and stress in patients suffering from Chronic Suppurative Otitis Media

**DOI:** 10.12669/pjms.35.2.152

**Published:** 2019

**Authors:** Shafaque Mehboob, SM Tariq Rafi, Naveed Ahmed, Mehjabeen

**Affiliations:** 1 *Shafaque Mehboob Khan, M.Phil, Lecture, Faculty of Pharmacy. Jinnah Sindh Medical University, Karachi, Pakistan*; 2 *Dr. Prof. SM Tariq Rafi, F.C.P.S & F.R.C.S. Vice Chancellor, Jinnah Sindh Medical University, Karachi, Pakistan*; 3 *Dr. Naveed Ahmed, MBBS. Post Graduate, Jinnah Post Graduate Center, Karachi, Pakistan*; 4 *Dr. Mehjabeen, PhD, Dean, Federal Urdu University of Arts, Science and Technology, Karachi, Pakistan*

**Keywords:** Chronic suppurative otitis media, Depression, Hearing loss, Pure tone audiometry

## Abstract

**Objective:**

To study the correlation of hearing loss with depression, anxiety and stress in patients suffering from chronic suppurative otitis media in local population of Pakistan.

**Methods:**

This is a cross-sectional study conducted from May to September 2018 at tertiary care hospital of Karachi. One hundred and twenty patients of chronic suppurative otitis media were divided into three groups: Group-1 (maintained on ciprofloxacin), Group-2 (maintained on co-amoxicillin) and Group-3 (did not subject to the treatment).The measurement of hearing loss was carried out by pure tone audiometry (PTA) and the depression, anxiety and stress were scored taking depression, anxiety and stress scale (DASS) as tool. To observe the effect of hearing loss on different groups one way ANOVA was applied and Spearman correlation was used to find correlation of depression with hearing loss.

**Results:**

There was no significant difference found for hearing loss and severity among the groups treated with ciprofloxacin, co-amoxicillin and not maintained on antibiotic therapy. Positive correlations found between hearing loss and depression, anxiety and stress in patients of the three groups.

**Conclusion:**

Depression induced by hearing loss as a result of CSOM in patients need to be monitored during and after treatment and scored so that can be treated by counseling and antidepressant (if required). Information regarding this topic on population of Pakistan will be helpful for health care takers and policy makers to manage mental stress with hearing loss in CSOM.

## INTRODUCTION

Chronic suppurative otitis media (CSOM) is a middle ear inflammation usually accompanied with infection, leads to perforation of tympanic membrane and prevalent up to 72 cases in 1000 inhabitant in developing countries. According to estimation over two billion dollars are being consumed to treat ear infection in United States of America.^1^ It causes several pathological changes in tympanic membrane resulting in effusion and cholesteatoma which interrupt the mechanical conduction of sound waves in patients of all age group.^2^ The reported audiometric data of several studies suggested that hearing loss progressively increases with the duration of CSOM.^3^

Many studies have focused the association of depression with hearing loss and CSOM one of the leading factors of decreased hearing capability across the world. Risk factors such as socio-economical, duration of disease and occupational stress influence the characteristic audiologic alteration in the patients of CSOM and also contribute in depression.^1^,^4^ Sensonary or auditory modification such as higher bilateral auditory threshold, activation of auditory cortex may explore the sensory modulation for depression associated in conductive and sensoneural hearing loss in patients of CSOM.^5^,^6^

A very interesting study conducted on tinnitus patients with hearing loss to evaluate the co-morbidities of depression, stress and anxiety, taking depression, anxiety and stress scale (DASS) as tool to determine the correlation of depression, stress and anxiety with hearing loss in tinnitus patients. Although different scales are available to measure depression but psychometric properties of DASS showed better separation in factor loadings as compare to other scales and its tripartite model of depression, anxiety and stress make it possible to differentiate these conditions.^7^

Unfortunately very few studies have been conducted on CSOM patients to focus the co-relation or association of depression with hearing loss all over the world but in local population of Pakistan no authentic data is available which satisfy the sample size of the study, therefore, our objective was to evaluate the correlation of hearing loss with depression, anxiety and stress in patients suffering from chronic suppurative otitis media in local population of Pakistan.

## METHODS

The present study was reviewed and approved by Institutional Review Board of Jinnah Sindh Medical University and conducted in ear, nose and throat department of a tertiary health care in accordance with the relevant regulations. This is a cross-sectional research on patients with the clinical presentation of CSOM subjected to PTA after evaluation of medical history and examination with otoscope.

### Inclusion criteria;

patients aged between 18-75 years, both the genders having unilateral ear presentation of CSOM without fluid discharge at the time of pure tone audiometry.

### Exclusion criteria;

paediatric population and patients above 75 years with the history of neurological disorder or profound psychological distress, cardiac arrest, family history of sensoneural hearing loss or using hearing aid.

Sample size calculation; the minimum sample size was calculated (n=68) with the help of OPEN-EPI, keeping 6.3% prevalence of CSOM and 95% confidence interval, taking 5% margin of error 1 but we enrolled 120 patients and divide them into three groups as group-1; treated with ciprofloxacin, group-2; treated with co-amoxicillin and group-3; untreated (control positive) to observe comparative effects of ciprofloxacin and co-amoxicillin on CSOM patients (40 patients of both gender per group).

PTA was examined at differences frequencies using an audiometer with aural headphones to measure hearing thresholds and divide into seven categories as normal (25 db), mild (26 to 34 db) HL, moderate (50-64 db) HL, severe (65-79 db) HL, profound (80 to 94 db) HL and deaf.^8^ In order to measure the negative emotions of depression, anxiety and stress, DASS was used as tool and Likert scale was taken for scoring.^7^

### Statistical analysis;

Associations between hearing loss and depression was evaluated by Spearman correlation test. The effect of hearing loss (HL) on different groups was compared (on both the genders) using one way ANOVA of independence (p< 0.01). In order to check the co-relation among different variables such as age, gender, hearing loss type (HLT) and hearing loss severity (HLS) Pearson correlation was applied.

## RESULTS

One hundred twenty patients of CSOM of both genders were equally divided into three groups in the current study as shown in Table-I.

**Table-I:** Age and hearing loss severity (HLS) are presented as mean with + S.D and gender and hearing loss type (HLT) as ratio. Correlation (Pearson) is applied to evaluate the significant value (*p<0.01).

Variables	Ciprofloxacin-group	Co-amoxicillin group	Untreated group	Correlation
No. of cases	40	40	40	–––––––––––––
Male/female ratio	1:1	1:1	1:1	Significant correlation of gender with HLT (P<0.01)
Age (mean)	39.80±11.20	43.25±9.8	45.3±8.7	Significant correlation of age with HLS (P<0.01)
Hearing loss type (%); mixed/conductive ratio	1.:1.66	1:1.85	1:2.07	insignificant correlation with HLS(P>0.01)
Hearing loss severity (db)	43.88±12.8	48.72±15.16	50.44±11.95	insignificant correlation with HLT (P>0.01)

No significant difference was observed for hearing loss severity and hearing loss type for groups as shown in Table-II.

**Table-II:** One way ANOVA for hearing loss type (HLT) & hearing loss severity (HLS) showing insignificant difference among all groups.

		Degree of freedom	Mean Square	F value	p≥.0.01
Hearing loss severity	Between Groups	3	4.267	2.468	0.066
	Within Groups	116	1.729		
Hearing loss type	Between Groups	3	.775	1.907	0.132
	Within Groups	116	.406		

**Fig.1:**
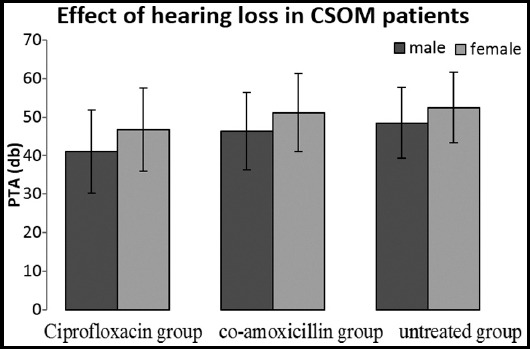
PTA of 1-3 groups in male and female CSOM patients. One way ANOVA showed insignificant differences among mentioned three groups (p<0.01).

The results of PTA with the corresponding groups are presented in mean±S.D in Fig.1, showing the higher hearing loss in female population as compare to the male. Although the patients without treatment showed higher hearing loss than treated groups and patients maintained on ciprofloxacin had better hearing threshold but no significant differences among all the groups as shown in Fig.1.

**Fig.2:**
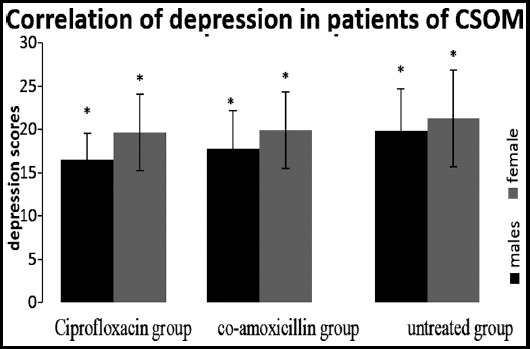
Spearman correlation showed positive correlation of both gender of group (ciprofloxacin), 2(co-amoxicillin) & 3(untreated) with depression induced by CSOM (*p<0.01).

**Fig.3:**
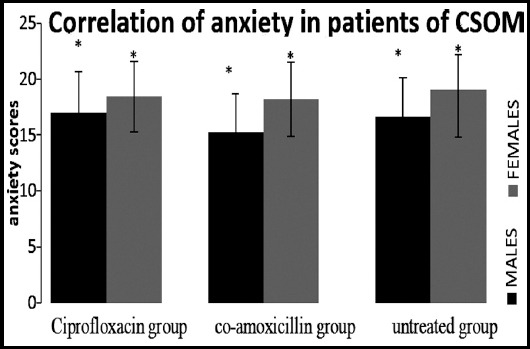
Spearman correlation showed positive correlation of both gender of group1 (ciprofloxacin), 2(co-amoxicillin) & 3(untreated) with anxiety induced by CSOM (*p<0.01).

**Fig.4:**
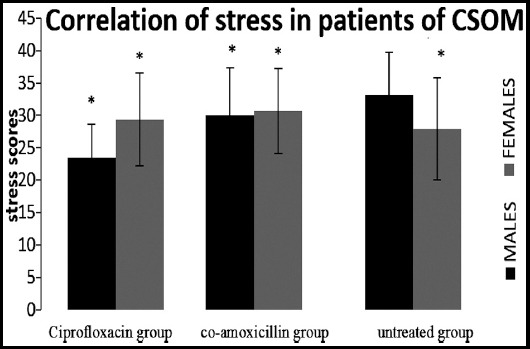
Spearman correlation showed positive correlation of both gender of group1 (ciprofloxacin), 2(co-amoxicillin) & 3(untreated) with stress induced by CSOM, (*p<0.01).

The positive correlation of depression, anxiety and stress with both the genders in CSOM patient was observed as shown in Fig. 2 to 4.

## DISCUSSION

In this study we had selected 120 patients of both gender in ratio of 1:1 of CSOM and divided them into three groups (40 patients per group) keeping prevalence rate of South Asia^1^ in view to the justified sample size.

Gender of the patients was significantly correlated with the hearing loss type (p=0.001) in our data and figure-1 showed that female patients suffered more hearing loss as compare to the male patients in the selected groups of patients. This finding is supported by the research in which role of hormones released from ovaries were also reviewed with other co-factors associated in females population which can damage hearing capabilities of women with aging and contribute to a poorer ability to hear.^9^

In our study significant correlation of age was observed with hearing loss severity (p=0.002) in our study and Table-1 displayed that the severity of hearing loss increased with the age of the patients. Therefore, our results are supported by the studies which presented the risk factor of adult patients who were more susceptible for hearing loss.^6^,^10^

Patients of Group-1 (maintained on ciprofloxacin) had average hearing threshold of 43.88 db, Group-2 (treated with co-amoxicillin) had 48.72 db and Group-3 (without any treatment) showed 50.44 db, poorer than normal threshold levels as reported in other studies conducted on clinical samples.^11^ The values showing hearing loss (HLS) were insignificantly correlate with type of hearing loss (conductive or mixed i-e, conductive with sensorineural hearing loss).

Although an earlier study showed that 13% cases of Sensorineural Hearing Loss found in CSOM^11^ patients but the association of all types of hearing loss (conductive, Sensorineural and mixed) with the severity of hearing loss indicating average threshold of CSOM patients is still not established. Therefore, our finding will be helpful to focus the attention of hearing healthcare policy makers to develop data highlighting all these facts and figures at larger cohort to make better and easier interpretations.^12^

Figure-1 showed that both the groups (maintained on ciprofloxacin and co-amoxicillin) displayed improved hearing threshold as compare to the diseased group which remained untreated but Group-I which was maintained on ciprofloxacin showed the best result which is supported by the previous studies^13^,^14^ but very interesting result was obtained when one way ANOVA was applied and there was no significant difference among the groups in hearing threshold values. This may be due to the insignificant improvement in middle ear perforation and temporal bone errosion size as demonstrated by earlier studies.^15^,^16^

Spearman’s correlation proved the positive correlation among different variables taken under consideration including hearing loss type (hlt), age and hearing loss severity (hls) with depression, anxiety and stress. In addition to this, female seemed to developed symptoms of depression and anxiety more than male as shown in figure-2 and 3 as reported in literature.^17^

The significant depression found in all three groups (evaluated on the interpretations of the responses given by the participants) showed the behavioral changes of the patients of CSOM due to the frustration with the condition of hopelessness, devaluation of life and lack of motivation. It was also observed in another observational study conducted to evaluate the association of hearing loss with cognition and depression on 5043 patients that hearing loss increased the risk of depression.^18^ Similarly, significant correlation of anxiety found in all groups showed the autonomic arousal and skeletal muscle affects due to hearing loss in CSOM patients as per interpretation of depression, anxiety and stress scale. In another correlation study on 7389 respondents’ high risk of depression along with anxiety syndrome appeared with hearing impairment.^19^ Stress also showed significant correlation with all the groups indicating the difficulty in relaxing nervous arousal and over all over-active reactions of the patients as supported by reported data.^4^

Therefore, we are agree with Hasson et al.^4^ which reflects the interpretation that hearing loss group developed stress but disagree with Gomaa et al.^7^ who highlighted that there is no interrelationship between hearing loss, its symptoms and severity with stress. Hence, patients with hearing loss due to CSOM are susceptible to develop depression.

### Limitation of the study

Data on depression, anxiety and stress was based on self-reported questionnaires which is limited to the past week experience based responses and majority of the respondents had low literacy profile. Patients with the family history of hearing loss or depression were not included.

## CONCLUSION

Depression induced by hearing loss as a result of CSOM in patients should be scored during and after treatment and risk factors should be focused so that can be treated by counseling and antidepressant (if required). It will guide the care takers to cure the patient mentally and physically. This study will help the health providers and researchers for the better management of the consequences of CSOM.

### Authors’ Contribution

**SMK** conceived, data collection, designed and manuscript writing.

**SMTR** did review and final approval of manuscript.

**NA** did data collection.

**M** editing of manuscript.
